# Electrocatalytic Properties of Quasi-2D Oxides LaSrMn_0.5_M_0.5_O_4_ (M = Co, Ni, Cu, and Zn) for Hydrogen and Oxygen Evolution Reactions

**DOI:** 10.3390/molecules29133107

**Published:** 2024-06-29

**Authors:** Kinithi M. K. Wickramaratne, Farshid Ramezanipour

**Affiliations:** Department of Chemistry, University of Louisville, Louisville, KY 40292, USA

**Keywords:** solid state oxide, quasi-2D, hydrogen evolution reaction, oxygen evolution reaction

## Abstract

Designing cost-effective and highly efficient electrocatalysts for water splitting is a significant challenge. We have systematically investigated a series of quasi-2D oxides, LaSrMn_0.5_M_0.5_O_4_ (M = Co, Ni, Cu, Zn), to enhance the electrocatalytic properties of the two half-reactions of water-splitting, namely oxygen and hydrogen evolution reactions (OER and HER). The four materials are isostructural, as confirmed by Rietveld refinements with X-ray diffraction. The oxygen contents and metal valence states were determined by iodometric titrations and X-ray photoelectron spectroscopy. Electrical conductivity measurements in a wide range of temperatures revealed semiconducting behavior for all four materials. Electrocatalytic properties were studied for both half-reactions of water-splitting, namely, oxygen-evolution and hydrogen-evolution reactions (OER and HER). For the four materials, the trends in both OER and HER were the same, which also matched the trend in electrical conductivities. Among them, LaSrMn_0.5_Co_0.5_O_4_ showed the best bifunctional electrocatalytic activity for both OER and HER, which may be attributed to its higher electrical conductivity and favorable electron configuration.

## 1. Introduction

The development of green and sustainable energy solutions is crucial to addressing the energy challenges of the future. The persistent use of carbon-rich fossil fuels leads to carbon dioxide emissions, worsening the greenhouse effect. Additionally, the harmful gases produced during the burning of those fuels cause environmental problems. These challenges have inspired the scientific community to explore renewable and sustainable energy sources. Hydrogen is widely considered an attractive alternative to conventional fuels due to its high energy density and absence of carbon emissions when used as a fuel. Among different methods of hydrogen production, electrochemical water splitting is highly promising. It is an excellent approach for converting and storing renewable energy if cost-effective and efficient catalysts are used to facilitate large-scale hydrogen production. Electrochemical water splitting involves two half-cell reactions, the hydrogen-evolution reaction (HER) and the oxygen-evolution reaction (OER). The OER also occurs in other energy storage and conversion devices, including rechargeable metal–air batteries (M_x_O_2_ → M_x_ + O_2_) [1]. The OER in water electrolysis involves a four-electron-transfer mechanism (H_2_O → O_2_ + 4H^+^ + 4e^−^) with a more complex reaction pathway and slower kinetics compared to the HER, which follows a two-electron-transfer mechanism (2H^+^ + 2e^−^ → H_2_). There are efficiency challenges associated with water electrolysis due to the sluggish kinetics of the two half-reactions, particularly the OER. This leads to substantial overpotentials beyond the theoretical thermodynamic potential of 1.23 V versus the reversible hydrogen electrode (RHE) [2]. Although catalysts based on noble metals, such as Pt, Ru, and Ir, often exhibit very high performance for water-splitting, their high cost and scarcity limit their wide-scale application, making it necessary to explore cost-effective and earth-abundant alternatives [3,4].

**Figure 1:**
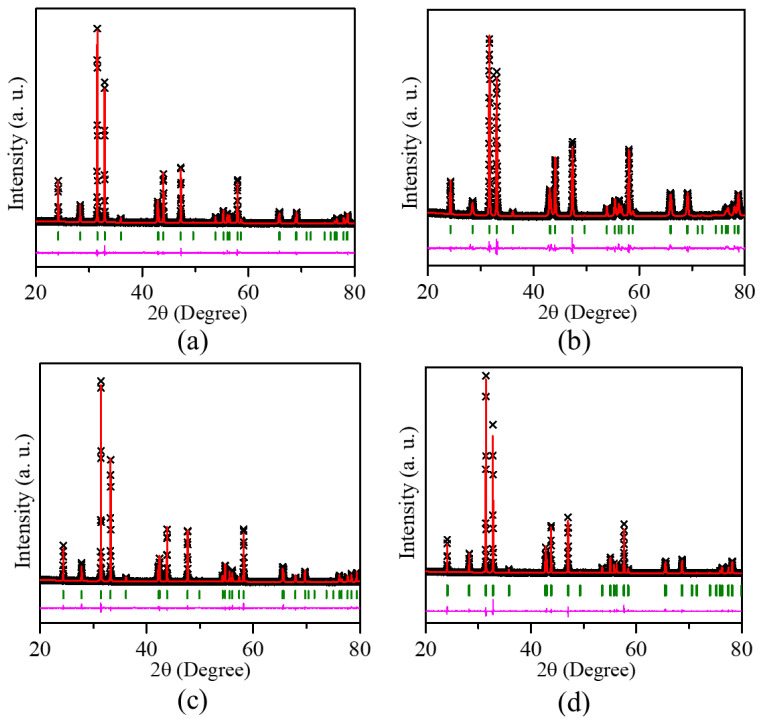
Rietveld refinement profiles using powder X-ray diffraction data of (**a**) LaSrMn_0.5_Co_0.5_O_4_, (**b**) LaSrMn_0.5_Ni_0.5_O_4_, (**c**) LaSrMn_0.5_Cu_0.5_O_4-δ_, and (**d**) LaSrMn_0.5_Zn_0.5_O_4_. The cross symbols, solid red line, olive vertical tick marks, and lower magenta line correspond to experimental data, the calculated pattern for the structural model, Bragg peak positions, and the difference plot, respectively.

Perovskite oxides have received growing interest as non-precious metal-based catalysts due to their versatility in composition, tunable electronic structures, affordability, and strong inherent catalytic performance [5,6,7,8]. Partial replacement or doping with different metal cations leads to changes in their performance for different applications driven by the resulting modifications to their physical, chemical, and electronic properties [9,10]. Several perovskite oxides, such as the benchmark catalyst Ba_0.5_Sr_0.5_Co_0.8_Fe_0.2_O_3−δ_ (BSCF), have demonstrated efficient OER catalysis, gaining prominence for their OER performance in alkaline environments, on par with IrO_2_ [5]. Studies have also explored the HER characteristics of perovskite oxides such as Pr_0.5_(Ba_0.5_Sr_0.5_)_0.5_Co_0.8_Fe_0.2_O_3-δ_ [11] and SrNb_0.1_Co_0.7_Fe_0.2_O_3-δ_ [12]. A variety of other structural arrangements are derived from perovskites, such as double perovskite, brownmillerite, Ruddlesden-popper, and the so-called K_2_NiF_4_-type [13] structure. The latter is the topic of the present work and has a quasi-2D structure (Figure 1) with the general formula A_2_BO_4_, where A is usually a rare-earth or alkaline-earth metal, and B is frequently a transition metal. Some materials with this structure type have been reported to show electrocatalytic activity for OER or HER, such as SrLaFeO_4_, SrLaCo_0.5_Fe_0.5_O_4_, and SrLaCoO_4-δ_ [13]. Nevertheless, oxide catalysts that demonstrate efficient catalytic activity for both OER and HER are not commonly found.

In the present work, we investigated a series of quasi-2D oxides LaSrMn_0.5_M_0.5_O_4_ (M = Co, Ni, Cu, Zn), with the aim of improving their electrocatalytic performance for HER and OER through the incorporation of different transition metals. Previous reports on these materials have been limited to their synthesis and some structural characterizations [14,15,16,17,18]. Their electrocatalytic properties have not been previously studied. Our comprehensive studies involved the investigation of their electrical and electrocatalytic properties, as well as detailed X-ray diffraction, X-ray photoelectron spectroscopy (XPS), and scanning electron microscopy (SEM) studies. Our findings showed semiconducting behavior in all four compounds, with electrical conductivity increasing as a function of temperature. The trend in electrical conductivity correlated with that of the electrocatalytic activity for both HER and OER. Importantly, the bifunctional electrocatalytic properties found in these materials are remarkable, particularly for LaSrMn_0.5_Co_0.5_O_4_, which exhibited the highest electrical charge-transport and superior electrocatalytic performance.

## 2. Results and Discussion

### 2.1. Crystal Structure

**Figure 2:**
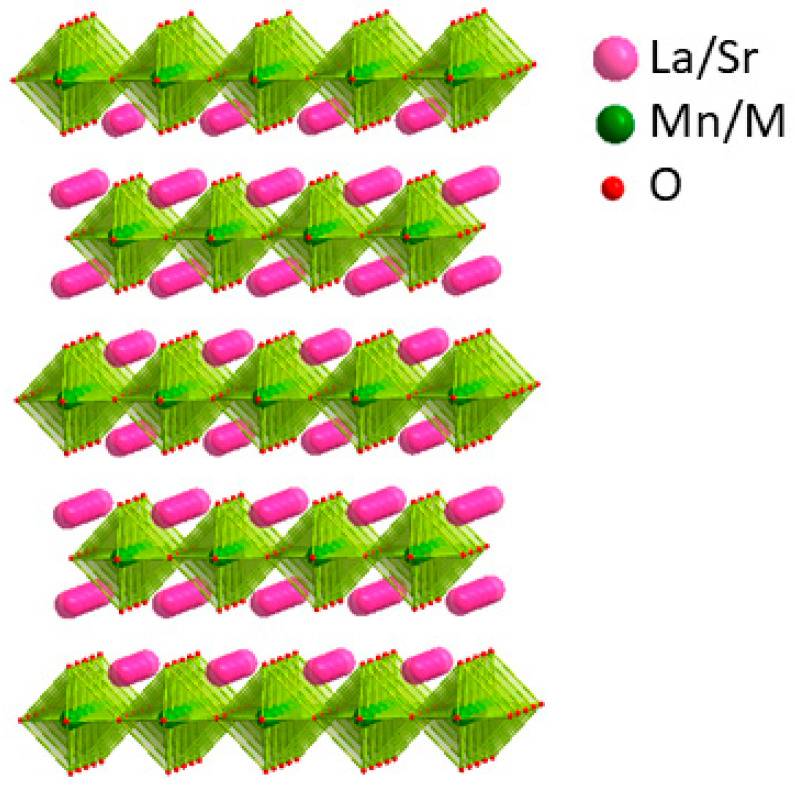
Crystal structure of LaSrMn_0.5_M_0.5_O_4_ (M = Co, Ni, Cu, Zn). Pink spheres denote La/Sr, small red spheres indicate oxygen, and green spheres represent Mn/M.

**Figure 3:**
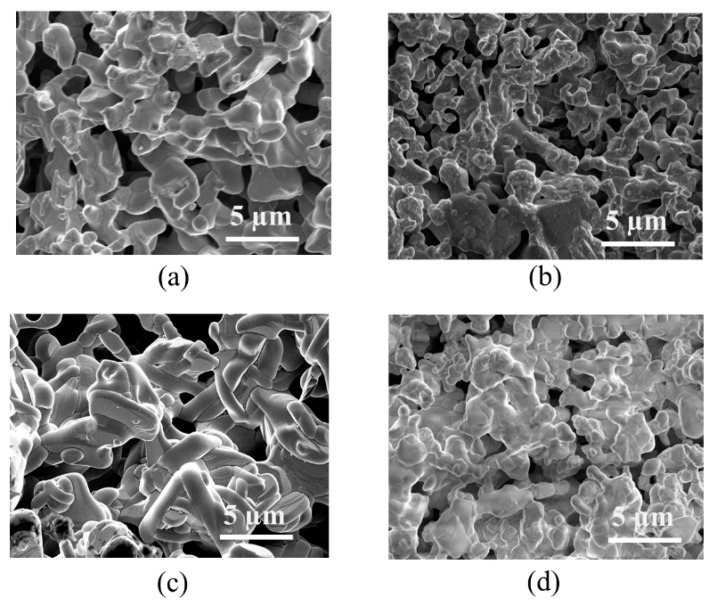
Scanning electron microscopy (SEM) images of (**a**) LaSrMn_0.5_Co_0.5_O_4_, (**b**) LaSrMn_0.5_Ni_0.5_O_4_, (**c**) LaSrMn_0.5_Cu_0.5_O_4-δ_, and (**d**) LaSrMn_0.5_Zn_0.5_O_4_.

Powder X-ray diffraction (XRD) was employed to assess the crystal structures of LaSrMn_0.5_M_0.5_O_4_ (M = Co, Ni, Cu, Zn) using Rietveld refinements (Figure 1). Table 1 shows a representative example of refined structural parameters for LaSrMn_0.5_Co_0.5_O_4_. The structural parameters for the other three materials are listed in Tables S1–S3. The data revealed that all samples consisted of a single phase, indicating the absence of impurities. The space group and structures of these materials were consistent with previous reports [14,15,16,17,18]. All four materials are isostructural with a body-centered tetragonal space group *I*4/*mmm*. The crystal structure is shown in Figure 2. All four materials can be described by the general formula A_2_BO_4_, where the A-site is occupied by a mix of La and Sr, while the B-site contains a mix of Mn with Co, Ni, Cu, or Zn. Their structures comprise BO_6_ octahedra that share corners to form a 2D layer. The layers are separated from each other, and the gap between them is where the A-site cations reside. The A-site cations have 9 oxygens in their coordination environment. The morphologies of sintered pellets were probed using scanning electron microscopy (SEM), and the results are shown in Figure 3, indicating similar microstructures for the four samples. The EDS mapping showed the surface elemental compositions, as shown in Figure S1, which illustrates representative data for LaSrMn_0.5_Co_0.5_O_4_, matching the expected atomic percentages.

### 2.2. Oxidation State and Oxygen Content

The synthesis conditions and elevated temperatures necessary for synthesizing these compounds can result in different valence states, which can affect the oxygen contents [19,20]. The oxygen contents of these materials were determined by iodometric titrations, which were repeated at least three times with excellent reproducibility, giving oxygen stoichiometries of 4.0173 ± 0.0016 for LaSrMn_0.5_Co_0.5_O_4_, 3.8530 ± 0.0073 for LaSrMn_0.5_Cu_0.5_O_4-δ_, 4.0039 ± 0.0105 for LaSrMn_0.5_Ni_0.5_O_4_, and 4.0120 ± 0.0164 for LaSrMn_0.5_Zn_0.5_O_4_. Therefore, iodometric titrations indicate four oxygens per formula unit for LaSrMn_0.5_Co_0.5_O_4_, LaSrMn_0.5_Ni_0.5_O_4,_ and LaSrMn_0.5_Zn_0.5_O_4_, matching the expected stoichiometries. However, LaSrMn_0.5_Cu_0.5_O_4-δ_ shows oxygen deficiency, with an oxygen stoichiometry of ~3.85 per formula unit (δ ≈ 0.15).

**Figure 4:**
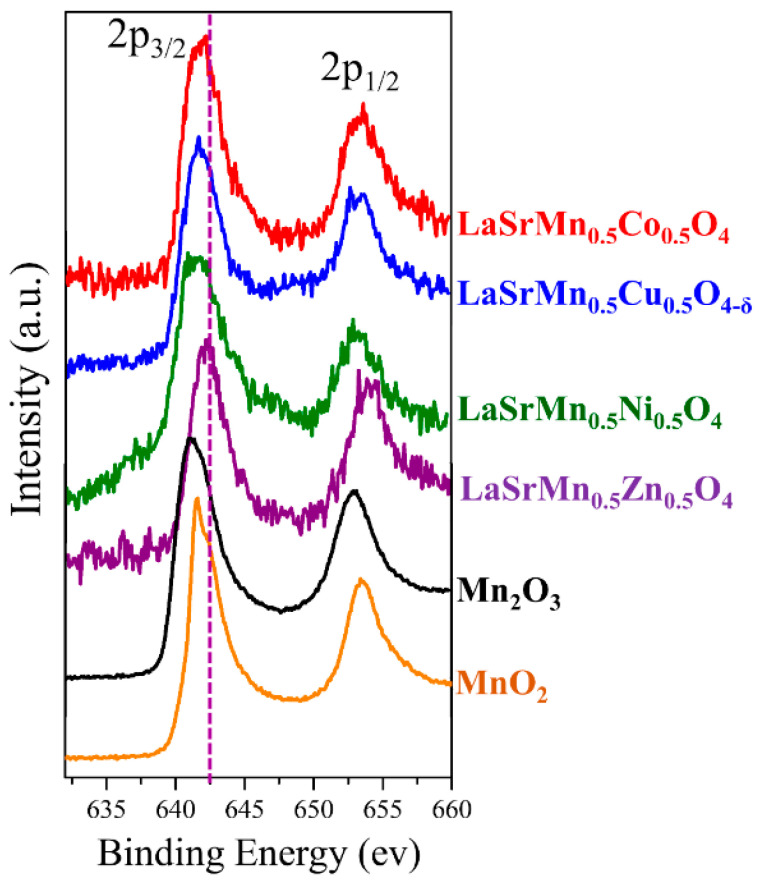
XPS data showing the manganese spectra for all four materials. The purple dashed line is drawn to show that the binding energy for the 2p_3/2_ peak of LaSrMn_0.5_Zn_0.5_O_4_ is shifted to higher energy compared to those of the other three materials.

We also investigated all four materials by X-ray photoelectron spectroscopy (XPS) to determine the oxidation states of transition metals. Given the close overlap of binding energies of Mn^3+^ and Mn^4+^ peaks, we also conducted additional measurements of standard Mn_2_O_3_ and MnO_2_ samples (Figure 4). The manganese spectra for three materials, LaSrMn_0.5_Co_0.5_O_4_, LaSrMn_0.5_Ni_0.5_O_4,_ and LaSrMn_0.5_Cu_0.5_O_4-δ_, are very similar to each other and encompass binding energies corresponding to both trivalent and tetravalent manganese, as evident from comparison to Mn_2_O_3_ and MnO_2_ standards in Figure 4 and previously reported manganese binding energies in other oxide materials [21,22,23]. Therefore, these three materials contain manganese in a mixed +3/+4 state. On the other hand, the manganese spectrum of LaSrMn_0.5_Zn_0.5_O_4_ shows peaks at a higher binding energy compared to the other three compounds, indicating a significantly greater tetravalent character. This observation is consistent with iodometric titration results that indicated four oxygens per formula unit. This oxygen content requires manganese in a tetravalent state, given the divalent state of zinc in LaSrMn_0.5_Zn_0.5_O_4_.

**Figure 5:**
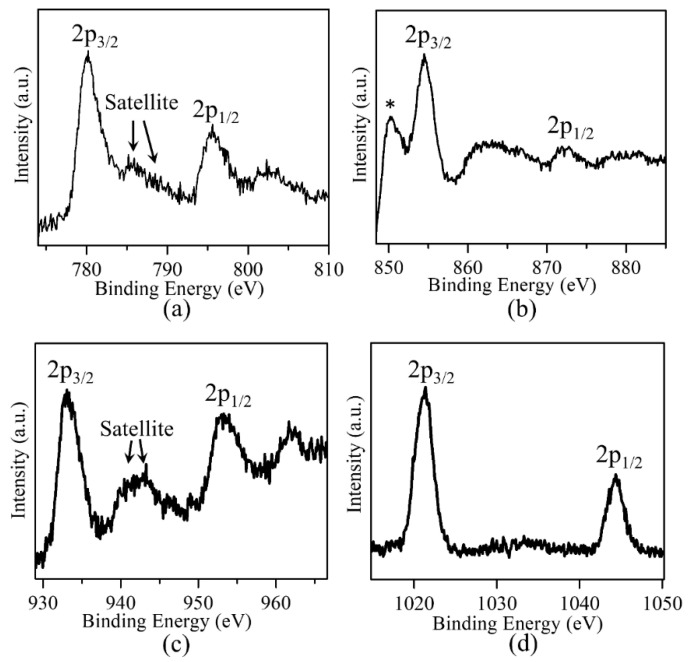
XPS data showing the spectra for (**a**) cobalt in LaSrMn_0.5_Co_0.5_O_4_, (**b**) nickel in LaSrMn_0.5_Ni_0.5_O_4_, (**c**) copper in LaSrMn_0.5_Cu_0.5_O_4–δ_, and (**d**) zinc in LaSrMn_0.5_Zn_0.5_O_4_. The peak marked by * is the lanthanum 3d_3/2_ peak, which appears very close to the nickel 2p_3/2_.

For LaSrMn_0.5_Co_0.5_O_4_, the cobalt spectrum was obtained (Figure 5a) and showed binding energies consistent with a mix of +2/+3 oxidation states. Divalent cobalt is expected to give a 2p_3/2_ peak just below 780 eV [24], while the peak for trivalent cobalt is expected to be just above 780 eV [24]. The Co 2p_3/2_ peak for our material is centered around 780 eV, with a satellite peak at about 786 eV, indicative of Co^2+^ [25], and a satellite at about 789 eV, indicative of Co^3+^ [24]. We ruled out the presence of tetravalent cobalt, as we did not observe any pronounced peak at ~281.5 eV [24]. These analyses are consistent with iodometric titration results, indicating four oxygens per formula unit for LaSrMn_0.5_Co_0.5_O_4_, and the manganese XPS spectrum, indicating manganese in mixed +3/+4 state, which would require cobalt to be in mixed +2/+3 state for the charge neutrality of the material.

For LaSrMn_0.5_Ni_0.5_O_4_, the nickel spectrum was obtained, as shown in Figure 5b. In this spectrum, the first peak, just above 850 eV, corresponds to La^3+^ 3d_3/2_ [26]. The 2p_3/2_ peak for Ni^2+^ is expected to appear close to 854 eV [26,27,28], but higher binding energies, such as ~855 eV, have also been reported for divalent nickel [29]. On the other hand, the Ni^3+^ 2p_3/2_ peak is expected to be close to 856–857 eV [26,27,29]. In our nickel spectra, the 2p_3/2_ peak is centered around ~254.6 eV, consistent with the predominant presence of Ni^2+^, and a small high-energy shoulder at ~856.5 eV, which may indicate the presence of some Ni^3+^.

For LaSrMn_0.5_Cu_0.5_O_4-δ_, the copper spectrum was obtained, as illustrated in Figure 5c. The Cu^2+^ 2p_3/2_ peak is expected to appear at ~933.5–934.6 eV [26,30,31,32], while the peak for Cu^1+^ should be close to ~932.4 eV [30,31,32]. In our copper spectra, the 2p_3/2_ peak is centered around ~933.7 eV, with strong satellite peaks at about 7–10 eV higher than the 2p_3/2_ peak, which is considered the signature of Cu^2+^ [31,33]. These observations are consistent with the presence of copper in a predominantly divalent state. They also match the observation of a Mn^3+^/Mn^4+^ mixture in the manganese spectrum and the oxygen vacancies determined by iodometric titrations, which will be required for charge neutrality.

For LaSrMn_0.5_Zn_0.5_O_4_, the zinc spectrum was obtained, as illustrated in Figure 5d. As expected, zinc is in divalent state, evident from the 2p3/2 peak at ~1021.3 eV [26,34]. As stated in the discussion of the manganese spectrum, the presence of Zn^2+^ and Mn^4+^ are consistent with iodometric titration results indicating four oxygens per formula unit.

### 2.3. Electrical Conductivity

We conducted a study on the electrical conductivity of all four compounds over a range of temperatures, from 25 to 800 °C (298 to 1073 K). The resistance is determined by applying Ohm’s law to the current response obtained from DC measurements, and this resistance value is subsequently converted into electrical conductivity, denoted by σ (Equation (1)) [35].

(1) $$\sigma =\frac{l}{RA}=\frac{I}{V}.\;\frac{l}{A}$$

**Figure 6:**
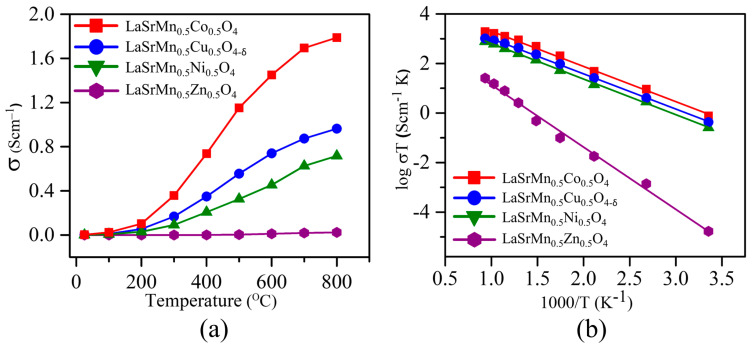
(**a**) Electrical conductivities as a function of temperature. (**b**) Arrhenius plots to determine the activation energies (E_a_) for the temperature-activated increase in conductivity.

In this equation, *l* indicates the thickness of the measured pellet, and *A* represents the cross-sectional area of the pellet through which the current is applied. Table 2 lists the conductivity values at room temperature, and Figure 6a shows the plot of conductivity at different temperatures.

As the temperature rises, the conductivities of all four materials increase, a behavior that is commonly observed in semiconducting materials. As temperature rises, charge carriers become more mobile, resulting in increased conductivity described by Equation (2).

(2) σ=neμ

In this equation, *σ* represents electrical conductivity, *n* denotes the concentration of charge carriers, *e* stands for charge of an electron, and *μ* represents the mobility of charge carriers [36,37]. LaSrMn_0.5_Co_0.5_O_4_ shows higher conductivity than the other three materials in the whole temperature range. The electrical conductivity in oxides typically occurs by electron/hole hopping through metal–oxygen–metal (M–O–M) bonds [38]. For this process to happen, metals with more than one stable oxidation state, such as Mn^3+^/Mn^4+^ or Co^3+^/Co^4+^, are preferred. The transition metal 3d orbitals overlap with oxygen 2p orbitals, allowing the electron hopping through the M–O–M pathway. The activation energy (E_a_) for the temperature-dependent increase in the conductivity of these materials can be calculated using the Arrhenius equation (Equation (3)) [35,39].

(3) $$\sigma T=\sigma^{0}e^{-\frac{E_{a}}{KT}}$$

The activation energy (E_a_) for the change in conductivity with temperature can be determined by analyzing the slope of the line of best fit in the log σT versus the 1000/T plot. In this equation, σ° is a pre-exponential factor, while E_a_, k, and T represent activation energy, the Boltzmann constant, and absolute temperature, respectively. Figure 6b shows the Arrhenius plots for the four materials. The activation energies, determined from the slopes, are presented in Table 2. It is important to emphasize that the activation energy signifies the energy barrier associated with the temperature-dependent increase in conductivity. The E_a_ values are consistent with the electrical conductivity trend, where LaSrMn_0.5_Co_0.5_O_4_, which exhibits higher conductivity, also has a lower activation energy.

### 2.4. Hydrogen Evolution Reaction

The hydrogen evolution reaction is the cathodic half-reaction of water electrolysis and involves a two-electron transfer process [6,40]. To overcome the sluggish reaction kinetics and accelerate the rate of the HER, it is necessary to apply an overpotential. Designing efficient and stable electrocatalysts is essential to improving the reaction kinetics and minimizing the overpotential. The mechanism of the HER involves a series of steps in both acidic and alkaline media [41]. Each condition has advantages and disadvantages. The efficiency of HER in acidic media is expected to be better than that of alkaline environments due to faster kinetics [42]. The source of the hydrogen adsorbed on the catalyst in acidic media is a proton, whereas in alkaline HER, the adsorbed hydrogen comes from breaking the bond in the H_2_O molecule [43], as shown below.

Volmer reaction (acidic): H_3_O^+^ + M + e^−^ ⇌ M − H * + H_2_O
Volmer reaction (alkaline): H_2_O + M + e^−^ ⇌ M − H * + OH
Heyrovsky reaction (acidic): M − H * + H_3_O^+^ + e^−^ ⇌ M + H_2_ + H_2_O
Heyrovsky reaction (alkaline): M − H * + H_2_O + e^−^ ⇌ M + H_2_ + OH^−^
Tafel reaction (acidic and alkaline): 2M − H * ⇌ 2M + H_2_

**Figure 7:**
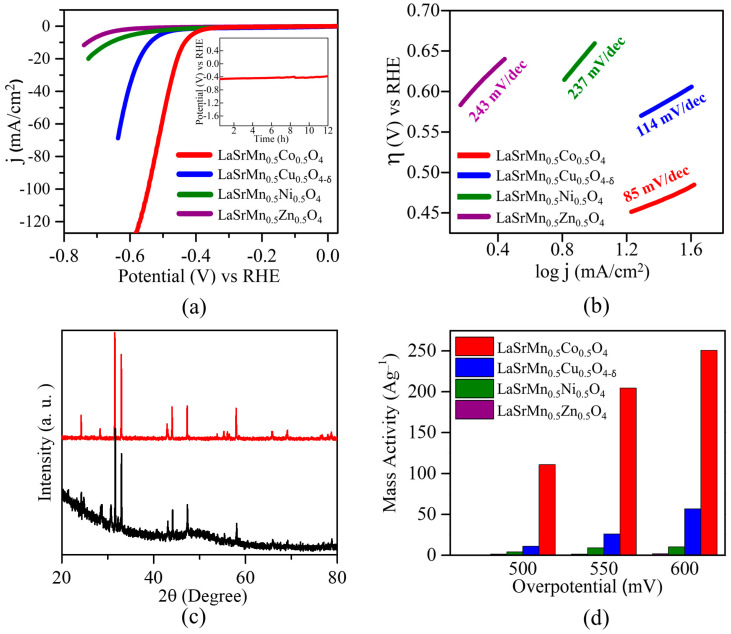
(**a**) Polarization curves for HER in 0.5 M H_2_SO_4_. The inset shows chronopotentiometry data for the best-performing material, LaSrMn_0.5_Co_0.5_O_4_. (**b**) Tafel plots and slopes. (**c**) X-ray diffraction data for LaSrMn_0.5_Co_0.5_O_4_ before and after 100 cycles of HER in 0.5 M H_2_SO_4_. (**d**) HER mass activities at different overpotentials in 0.5 M H_2_SO_4_.

Alkaline electrolysis may be favored in an industrial setting [44], due to some limitations of acidic conditions, such as problems with the stability of metal oxides in acids [13]. In this work, the HER experiments were performed in both alkaline (1 M KOH) and acidic (0.5 M H_2_SO_4_) conditions. In an acidic medium (Figure 7a), among the four materials, LaSrMn_0.5_Co_0.5_O_4_ shows the lowest overpotential of *η*_10_ = 427 mV at 10 mA/cm^2^, which is a current density used by convention and corresponds to a 12.3% solar-to-hydrogen efficiency. It is followed by LaSrMn_0.5_Cu_0.5_O_4-δ_ showing *η*_10_ = 535 mV, LaSrMn_0.5_Ni_0.5_O_4_ with *η*_10_ = 647 mV, and LaSrMn_0.5_Zn_0.5_O_4_ with *η*_10_ = 724 mV. Therefore, the HER activities show the following order: LaSrMn_0.5_Zn_0.5_O_4_ < LaSrMn_0.5_Ni_0.5_O_4_ < LaSrMn_0.5_Cu_0.5_O_4-δ_ < LaSrMn_0.5_Co_0.5_O_4_. The acidic HER overpotential of LaSrMn_0.5_Co_0.5_O_4_ is not as low as those observed in some other catalysts such as MoO_3-y_ nanofilms (*η*_10_ = 0.201 V) [45], WO_3_ nanoplates (*η*_10_ = 0.117 V) [46], and LaCa_2_Fe_3_O_8_ (*η*_10_ = 0.400 V) [47]. However, it is lower than those reported for some other oxides in acidic media, such as perovskite LaFeO_3_ (*η*_10_ = 0.490 V) [48], Ruddlesden-Popper oxide Sr_2_LaCoMnO_7_ (*η*_10_ = 0.612 V) [49], bilayered brownmillerite Ca_3_Fe_2_MnO_8_ (*η*_10_ = 0.507 V) [21], and K_2_NiF_4_-type oxide SrLaCoO_4-δ_ (*η*_10_ = −0.547 V) [13].

To explore the reaction kinetics for the four materials, the Tafel equation was used (Equation (4)) [50,51].

(4) η=a+b logj

where *η* is the overpotential and *j* is the current density. The slope of *η* vs. log*j* is indicative of the reaction kinetics. A smaller slope suggests a faster reaction, as it indicates that a small change in overpotential is associated with a large change in current density [51,52]. As shown in Figure 7b, the Tafel slopes of the four materials show the same trend as their overpotentials. LaSrMn_0.5_Co_0.5_O_4_ shows the smallest Tafel slope among the four compounds, indicating faster reaction kinetics and consistent with its greater electrocatalytic activity. Chronopotentiometry experiments performed on the most active material, LaSrMn_0.5_Co_0.5_O_4_, exhibit a stable response for over 12 h, as shown in the inset of Figure 7a. Furthermore, X-ray diffraction data, before and after 100 cycles of HER (Figure 7c), indicates little change, confirming that the material’s structural integrity remains unaltered. We also calculated the mass activity to evaluate the catalysts’ inherent qualities. In Figure 7d, we have compared the mass activities of the four compounds at various overpotentials, showing that LaSrMn_0.5_Co_0.5_O_4_ has a consistently higher mass activity.

**Figure 8:**
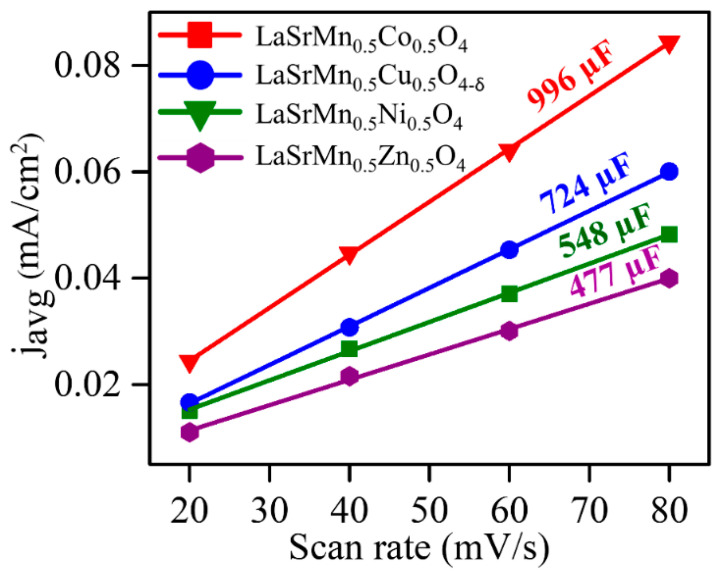
Plots of j_avg_ against ν in 0.5 M H_2_SO_4_. The slopes indicate double-layer capacitance, C_dl_.

We also determined the double-layer capacitance, C_dl_, (Figure 8) by analyzing the average current densities (j_avg_) at various scan rates (υ), as obtained from cyclic voltammograms in a non-faradaic region (Figure S2) [53]. The importance of C_dl_ is that it is proportional to the electrochemically active surface area [54]. The slope of the plot depicting j_avg_ against ν provides the double-layer capacitance, calculated according to Equation (5) [6].

j_avg_ = C_dl_ × ν (5)

Notably, the C_dl_ values align with the trend observed in electrocatalytic activity, with the most active catalyst, LaSrMn_0.5_Co_0.5_O_4_, also displaying the highest C_dl_ value.

**Figure 9:**
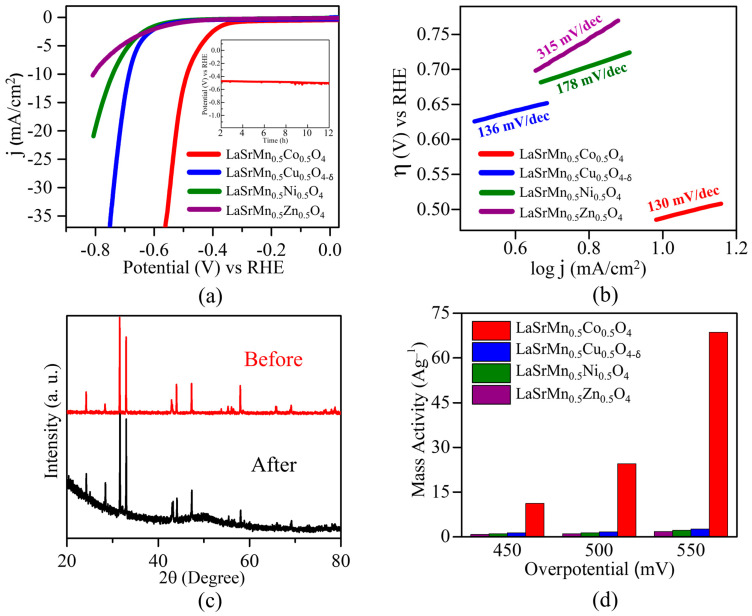
(**a**) HER polarization curves in 1 M KOH. The inset shows chronopotentiometry data for the best-performing material LaSrMn_0.5_Co_0.5_O_4_. (**b**) Tafel plots and slopes. (**c**) X-ray diffraction data for LaSrMn_0.5_Co_0.5_O_4_ before and after 100 cycles of HER in 1 M KOH. (**d**) HER mass activities at different overpotentials in 1 M KOH.

The HER activities of the four materials in basic conditions, 1 M KOH, show a similar trend to that in acidic conditions, where LaSrMn_0.5_Co_0.5_O_4_ has the best activity, followed by LaSrMn_0.5_Cu_0.5_O_4-δ_, LaSrMn_0.5_Ni_0.5_O_4_, and LaSrMn0_.5_Zn_0.5_O_4_ (Figure 9a). The overpotential values (η_10_) at 10 mA/cm^2^ are 0.482 V, 0.680 V, 0.740 V, and 0.805 V, respectively. Similarly, the same trend is observed for the HER kinetics, as evident from the Tafel slopes shown in Figure 9b. XRD data, before and after 100 HER cycles (Figure 9c), indicate that the structure remains unchanged, confirming the stability. In addition, the comparison of mass activity at various overpotentials in the basic medium again shows consistently higher mass activity for LaSrMn_0.5_Co_0.5_O_4_ (Figure 9d).

**Figure 10:**
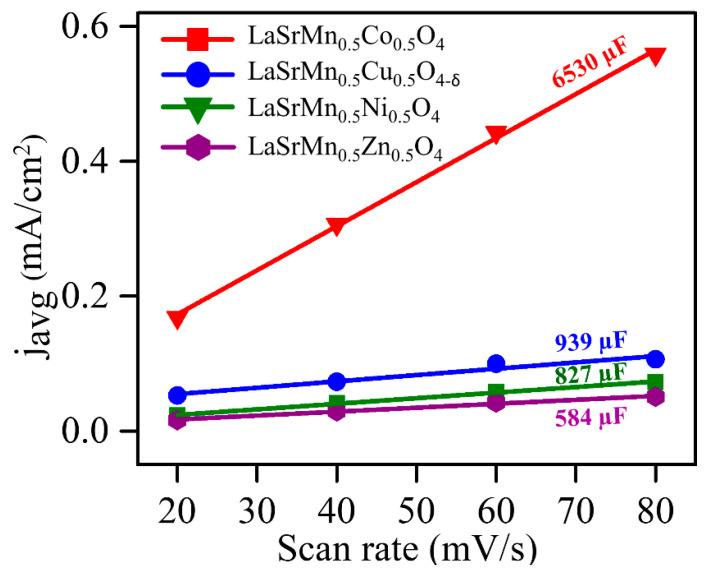
Plots of j_avg_ against ν in 1 M KOH. The slopes indicate double-layer capacitance, C_dl_.

We also determined the double-layer capacitance (C_dl_) in 1 M KOH (Figure 10) using cyclic voltammograms in the non-faradaic region (Figure S2). Among the four materials, LaSrMn_0.5_Co_0.5_O_4_ displays the highest C_dl_, followed by LaSrMn_0.5_Cu_0.5_O_4-δ_, LaSrMn_0.5_Ni_0.5_O_4_, and LaSrMn0_.5_Zn_0.5_O_4_, the same trend as the electrocatalytic activity.

### 2.5. Oxygen Evolution Reaction

**Figure 11:**
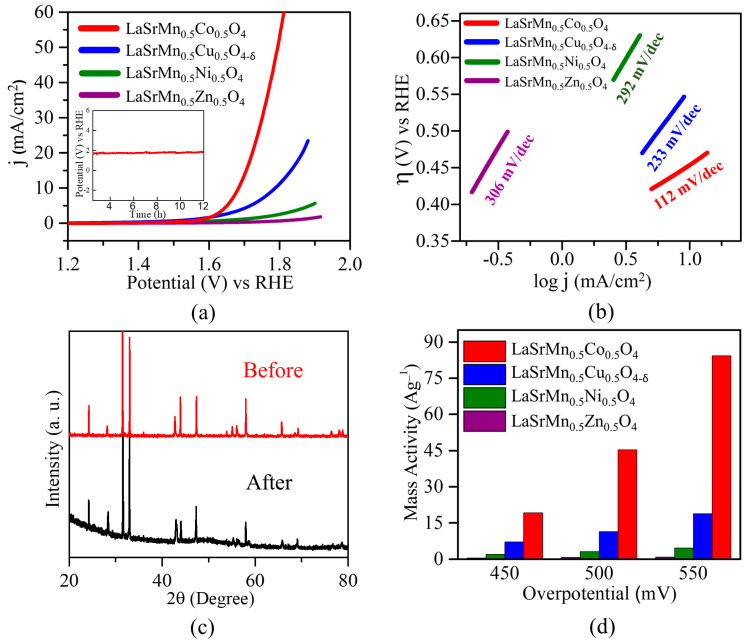
(**a**) Polarization curves for OER in 1 M KOH. The inset shows chronopotentiometry data for the best-performing material, LaSrMn_0.5_Co_0.5_O_4_. (**b**) Tafel plots and slopes. (**c**) X-ray diffraction data for LaSrMn_0.5_Co_0.5_O_4_ before and after 100 cycles of OER in 1 M KOH. (**d**) OER mass activities at different overpotentials in 1 M KOH.

The OER electrocatalytic activities of the four materials were studied in both acidic and alkaline media. These materials showed little OER activity in acidic conditions, but the alkaline OER activity, especially for two of the materials, was substantial. Therefore, alkaline OER is discussed here. As demonstrated by the polarization curves in Figure 11a, the electrocatalytic performance for OER follows a similar trend to that observed in the HER. Among the four compounds, LaSrMn_0.5_Ni_0.5_O_4_, and LaSrMn0_.5_Zn_0.5_O_4_ did not produce sufficient current to reach 10 mA cm^−2^ within the experimental potential range. On the other hand, LaSrMn_0.5_Cu_0.5_O_4-δ_ shows an overpotential of *η*_10_ = 550 mV, beyond the thermodynamic potential of 1.23 V at 10 mA cm^−2^, while LaSrMn_0.5_Co_0.5_O_4_ exhibits a lower overpotential of *η*_10_ = 450 mV. The OER overpotential of LaSrMn_0.5_Co_0.5_O_4_ (η_10_ = 450) is not as low as those observed for some oxides such as IrO_2_ (400 mV) and RuO_2_ (η_10_ ≈ 420 mV) [49]. Nevertheless, LaSrMn_0.5_Co_0.5_O_4_ shows a better activity than several previously reported oxides, including well-known catalyst BSCF (η_10_ ≈ 500 mV) [5], La_0.5_Sr_0.5_Co_0.8_Fe_0.2_O_3_ (η_10_ = 600 mV) [55], and La_0.6_Sr_0.4_CoO_3−δ_ (η_10_ = 590 mV) [56].

**Figure 12:**
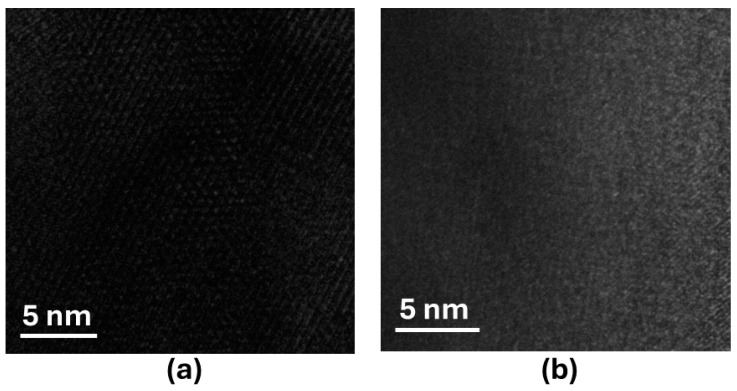
The TEM data (**a**) before and (**b**) after chronopotentiometry experiment under OER conditions.

The kinetics of the OER were analyzed using Tafel plots (Figure 11b), indicating a smaller Tafel slope, i.e., faster reaction kinetics, for LaSrMn_0.5_Co_0.5_O_4_ compared to the other three materials and the same trend as the OER activity. The stability of the best catalyst in the series, LaSrMn_0.5_Co_0.5_O_4_, was evaluated by chronopotentiometry, showing a steady response for at least 12 h, as shown in the inset of Figure 11a. Also, X-ray diffraction data after 100 cycles of OER confirmed the material’s structural integrity was preserved (Figure 11c). The mass activities for OER at various overpotentials are shown in Figure 11d, highlighting the significantly higher activity of LaSrMn_0.5_Co_0.5_O_4_. Figure 12 shows the TEM images before and after the chronopotentiometry experiment under OER conditions for LaSrMn_0.5_Co_0.5_O_4_.

Some comments on the observed electrocatalytic properties are in order. The trend in electrocatalytic activity matches the trend in electrical conductivity. It is important to note that both OER and HER involve the transfer of electrons and are affected by the conductivity of catalysts. Some previous studies have shown that perovskite oxides with better electronic conductivity exhibit improved electrocatalytic performance [6,36].

There are also other parameters to consider. Some researchers have suggested that the filling of e_g_-level orbitals can be a descriptor for the OER activity [5]. They proposed that high OER activity is expected for oxides, containing metal cations that have e_g_ occupancy close to one, due to the optimum binding with reaction intermediates. In the best catalyst in our series, LaSrMn_0.5_Co_0.5_O_4_, a mixture of Co^2+^ and Co^3+^ oxidation states, is observed. Previous studies have indicated that the intermediate spin state is prominent for Co^3+^ in some perovskite oxides [5,57]. This intermediate spin state would give an electron configuration of t_2_g^5^ e_g_^1^ for Co^3+^. Also, the mixed oxidation states of transition metals can accelerate OER activity [1,43], an advantage offered by the presence of cobalt. Regarding the next best material in the series, LaSrMn_0.5_Cu_0.5_O_4-δ_, it is possible that the observed activity is influenced by the presence of oxygen vacancies in this material, as confirmed by iodometric titrations and XPS, which might facilitate the adsorption of reaction intermediates [6,47,58]. In addition, some studies on HER catalysts containing both copper and nickel have shown the better activity of copper compared to nickel. For example, a study of NiCu showed that surface Cu exhibits a d-band center closer to that of the highly active catalyst Pt [59]. The Ni site of NiCu was found to play a less substantial role in HER, primarily due to its d-band being too high, while Cu emerged as the more active catalytic site [59,60]. Finally, the least active material in the series, LaSrMn0_.5_Zn_0.5_O_4_, has the lowest electrical conductivity and contains zinc, which is only in a divalent state.

## 3. Experimental Methods

Synthesis and Characterization. Polycrystalline samples of LaSrMn_0.5_Co_0.5_O_4_, LaSrMn_0.5_Ni_0.5_O_4_, LaSrMn_0.5_Cu_0.5_O_4-δ_, and LaSrMn_0.5_Zn_0.5_O_4_ were synthesized under an argon atmosphere by solid-state synthesis method. The stoichiometric proportions of precursors La_2_O_3_, SrCO_3_, MnO_4_, CoO, NiO, CuO, and ZnO were thoroughly mixed in agate mortar and pestle. For example, for the synthesis of LaSrMn_0.5_Co_0.5_O_4_, a mixture of La_2_O_3_ (0.1500 g), SrCO_3_ (0.1359 g), CoO (0.0343 g), and MnO_2_ (0.0400 g) was used. The powder mixtures were pressed into pellets and were heated at 1200 °C for 24 h followed by slow cooling. The heating and cooling rates of the furnace for all samples were set at 100 °C/h. The phase purity and structure of polycrystalline samples were determined by powder X-ray diffraction (XRD) at room temperature using Cu Kα1 radiation (λ = 1.54056 Å) on an X-ray diffractometer equipped with a monochromator. Rietveld refinements were carried out using GSAS [61] program and EXPIGU interface [62]. The oxygen content was determined using iodometric titrations [63], where excess potassium iodide (2 g) and 50 mg of the sample were dissolved in 100 mL of argon-purged 1 M HCl, allowing the mixture to react overnight. Then, 5 mL of the reacted mixture, containing the generated iodine, was titrated against 0.025 M Na_2_S_2_O_3_ (I_2_ + 2S_2_O_3_^2−^ → 2I^−^ + S_4_O_6_^2−^) using starch indicator (0.6 mL), which was added near the titration endpoint. Excess KI reduced the oxide samples to form oxides with the lowest stable oxidation states of transition metals. The amount of Na_2_S_2_O_3_ needed for titration indicated the amount of oxygen lost during metal ion reduction. Measurements were repeated three times for error analysis. The morphologies of the samples were studied by Scanning Electron Microscopy (SEM) on sintered pellets using a Thermo Fisher Apreo C LoVac Field Emission SEM at a magnification of 5000×, accompanied by an energy-dispersive X-ray spectroscopy (EDS) detector. X-ray photoelectron spectroscopy (XPS) measurements were performed using a ThermoFisher Scientific K-Alpha instrument. The instrument employed an aluminum monochromatic X-ray source with an energy of hν = 1486.69 eV and incorporated an electron flood gun for effective charge neutralization. Wide survey scans were carried out at a pass energy of 160 eV, while high-resolution scans were performed with a pass energy of 20 eV.

Electrical Conductivity. Electrical conductivity measurements were conducted at variable temperatures from 25 °C to 800 °C. These measurements were performed using a two-probe direct current (DC) method [64] on pellets that had been sintered at 1250 °C. Prior to conducting the measurements, a layer of gold paste was applied to both sides of the pellet and then dried by heating for 3 h at 800 °C. Gold wires were attached to gold foils and used as electrodes, ensuring contact with the two gold-painted surfaces on each side of the pellet. A voltage of 0.01 V was applied for the measurements, which were conducted at approximately 100 °C intervals. Equilibrium conductivity was achieved at each measurement temperature after about 30 min, as indicated by a stable plateau in the DC conductivity data. Heating and cooling rates during the conductivity measurements were maintained at 3 °C/min.

Electrochemical Measurements. The catalyst ink for electrochemical measurements was prepared using 35 mg of the catalyst material, 7 mg of carbon black powder, 40 μL of Nafion D-521 solution (5% *w*/*w* in water and 1-propanol), and 7 mL of Tetrahydrofuran (THF). The mixture was ultrasonically dispersed in water for 30 min. The drop-casting was carried out by placing two coats of 10 μL of the mixture onto the surface of a glassy carbon electrode (GCE) with a diameter of 5 mm and an area of 0.196 cm^2^, with a mass loading of 1.02 mg/cm^2^, followed by overnight air-drying. This catalyst-loaded electrode was used as the working electrode. Electrochemical measurements were conducted using a standard three-electrode electrochemical cell connected to a rotating disk electrode at 1600 rpm. The OER and HER experiments were carried out in 1 M KOH solution by using a Hg/HgO reference electrode (1 M NaOH). The HER experiments were also conducted in 0.5 M H_2_SO_4_ solution using Ag/AgCl (4 M KCl) as the reference electrode. A carbon electrode was used as the counter electrode for all experiments. All potentials were iR-corrected and converted to potential vs. reversible hydrogen electrode (RHE) using the Nernst equation *E*_*vsRHE*_ = *E*_*vs* Reference electrode_ + 0.059 pH + *E*^0^_Reference electrode_, where *E*^0^_*Ag*/*AgCl*_ = 0.197 V for 4 M KCl and *E*^0^_Hg/HgO_ = 0.098 for 1 M NaOH [36,65]. For each material, the electrocatalytic measurements were repeated at least three times, using at least two different batches synthesized independently. Chronopotentiometry was utilized to investigate the catalyst stability under both HER and OER conditions, employing the same three-electrode setup and a constant current of 10 mA/cm^2^.

## 4. Conclusions

A range of parameters can influence electrocatalytic properties, such as electrical conductivity, the presence of metals with more than one stable oxidation state, oxygen-vacancies, and electron configuration. These parameters were studied in this work through the investigation of a series of quasi-2D oxides to reveal their structural and electrocatalytic properties. X-ray diffraction, X-ray photoelectron spectroscopy, iodometric titrations, electrical conductivity studies, and OER and HER experiments were used for the investigation of the four materials LaSrMn_0.5_M_0.5_O_4_ (M = Co, Ni, Cu, and Zn). The electrical conductivity measurements, as well as electrocatalytic HER and OER, all showed the same trend, LaSrMn_0.5_Zn_0.5_O_4_ < LaSrMn_0.5_Ni_0.5_O_4_ < LaSrMn_0.5_Cu_0.5_O_4-δ_ < LaSrMn_0.5_Co_0.5_O_4_. The best-performing compound in this series, LaSrMn_0.5_Co_0.5_O_4_, can act as a bifunctional catalytic material, facilitating both OER and HER. Its lower overpotential, faster kinetics, and greater mass activity were demonstrated and correlated with several descriptors that can be responsible for its higher electrocatalytic performance.

## Figures and Tables

**Table 1:** Refined structural parameters for LaSrMn_0.5_Co_0.5_O_4_ at room temperature using powder X-ray diffraction data. Space group: *I*4/*mmm*, a = 3.8435 (1) Å, c = 12.5933 (2) Å, R_p_ = 0.0392, wR_p_ = 0.0504.

Atom	x	y	z	Occupancy	Multiplicity	U_iso_ (Å^2^)
La	0	0	0.35985 (8)	0.5	4	0.0192 (5)
Sr	0	0	0.35985 (8)	0.5	4	0.0192 (5)
Mn	0	0	0	0.5	2	0.0131 (9)
Co	0	0	0	0.5	2	0.0131 (9)
O1	0.5	0	0	1	4	0.031 (2)
O2	0	0	0.1642 (6)	1	4	0.038 (2)

**Table 2:** Room temperature electrical conductivity and activation energies.

	Electrical Conductivity (S/cm)	Activation Energy (eV)
LaSrMn_0.5_Co_0.5_O_4_	0.0026	0.2673 (2)
LaSrMn_0.5_Ni_0.5_O_4_	0.0014	0.2746 (2)
LaSrMn_0.5_Cu_0.5_O_4-δ_	8.5961 × 10^−4^	0.2676 (3)
LaSrMn_0.5_Zn_0.5_O_4_	7.0755 × 10^−4^	0.4727 (7)

## Data Availability

Data that support the findings of this study are available from the corresponding author upon reasonable request.

## Supplementary Materials

The following supporting information can be downloaded at: https://www.mdpi.com/article/10.3390/molecules29133107/s1, Figure S1: SEM images and corresponding EDS; Figures S2 and S3: Cyclic voltammetry data in non-faradaic region in 0.5 M H_2_SO_4_ and 1 M KOH, respectively; Tables S1–S3: Refined structural parameters.
