# Supplementary material for: Electrocatalytic Properties of Quasi-2D Oxides LaSrMn0.5M0.5O4 (M = Co, Ni, Cu, and Zn) for Hydrogen and Oxygen Evolution Reactions
Source: Molecules. 2024 Jun 29;29(13):3107. doi: 10.3390/molecules29133107 (PMC11243240; doi:10.3390/molecules29133107)
Supplement: Supplementary file 1 [file molecules-29-03107-s001.zip › molecules-3017740-supplementary.pdf]

Supporting Information

Electrocatalytic Properties of Quasi-2D Oxides $LaSrMn_{0.5}M_{0.5}O_4$ (M = Co, Ni, Cu, and Zn) for Hydrogen and Oxygen Evolution Reactions

Kinithi M. K. Wickramaratne[a], Farshid Ramezanipour[a,*]

[a]Department of Chemistry, University of Louisville, Louisville, Kentucky 40292, USA
*Corresponding author. Email: farshid.ramezanipour@louisville.edu, Phone: (502) 852-7061

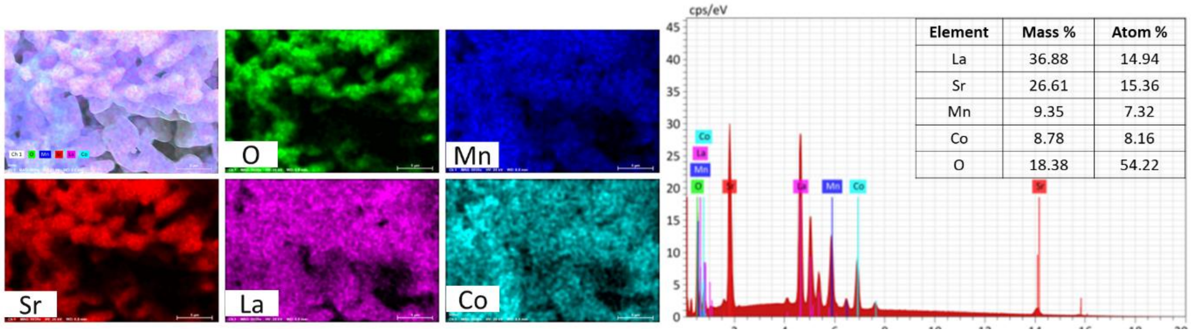

Figure S1. Scanning electron microscopy (SEM) images and the corresponding EDS mapping for $LaSrMn_{0.5}Co_{0.5}O_4$.

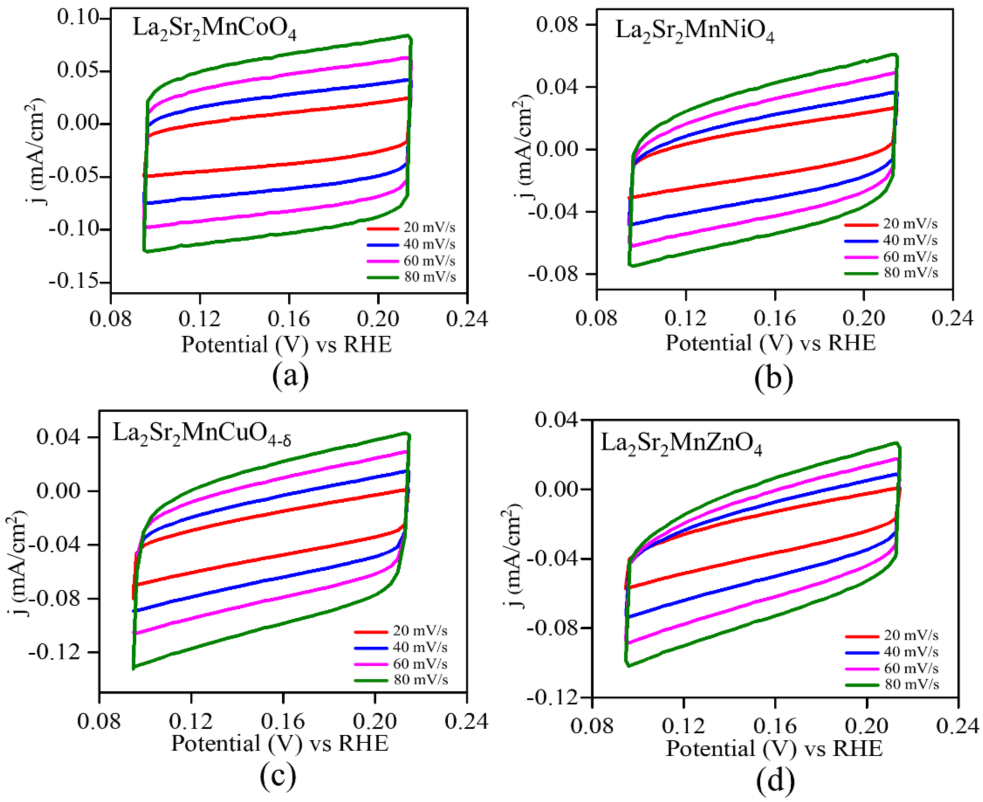

Figure S2. Cyclic voltammetry data in the non-faradaic region in 0.5 M H_2SO_4.

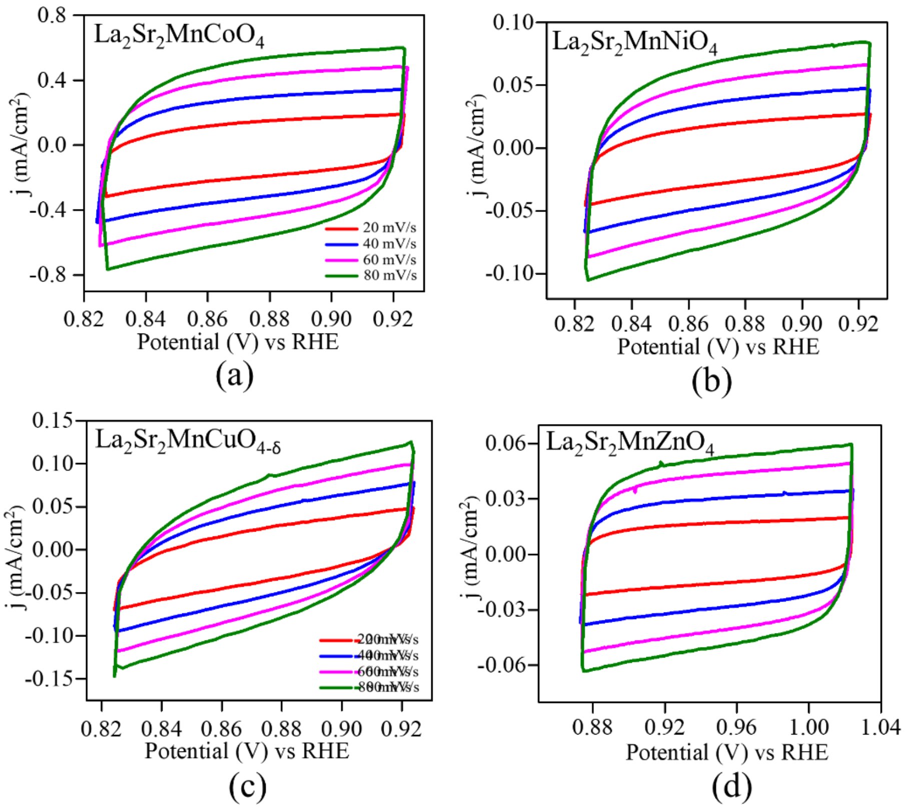

Figure S3. Cyclic voltammetry data in the non-faradaic region in 1 M KOH.

Table S1. Refined structural parameters for $LaSrMn_{0.5}Ni_{0.5}O_4$ at room temperature using powder X-ray diffraction data. Space group: *I4/mmm*, *a* = 3.8409(1) Å, *c* = 12.5694(3) Å, R_p = 0.0529, wR_p = 0.0714.

Atom	x	y	z	Occupancy	Multiplicity	U_{iso} (Å^2)
La	0	0	0.3597(1)	0.5	4	0.0283(5)
Sr	0	0	0.3597(1)	0.5	4	0.0283(5)
Mn	0	0	0	0.5	2	0.0224(9)
Ni	0	0	0	0.5	2	0.0224(9)
O1	0.5	0	0	1	4	0.028(2)
O2	0	0	0.1604(6)	1	4	0.032(2)

Table S2. Refined structural parameters for $LaSrMn_{0.5}Cu_{0.5}O_{4-\delta}$ at room temperature using powder X-ray diffraction data. Space group: *I4/mmm*, *a* = 3.8139(1) Å, *c* = 12.8459(3) Å, R_p = 0.0555, wR_p = 0.0721.

Atom	x	y	z	Occupancy	Multiplicity	U_{iso} (Å^2)
La	0	0	0.35906(7)	0.5	4	0.0128(5)
Sr	0	0	0.35906(7)	0.5	4	0.0128(5)
Mn	0	0	0	0.5	2	0.0101(9)
Cu	0	0	0	0.5	2	0.0101(9)
O1	0.5	0	0	0.98125	4	0.017(2)
O2	0	0	0.1659(5)	0.98125	4	0.036(2)

Table S3. Refined structural parameters for $LaSrMn_{0.5}Zn_{0.5}O_4$ at room temperature using powder X-ray diffraction data. Space group: *I4/mmm*, *a* = 3.8628(1) Å, *c* = 12.6301(1) Å, R_p = 0.0549, wR_p = 0.0706.

Atom	x	y	z	Occupancy	Multiplicity	U_{iso} (Å^2)
La	0	0	0.35982(7)	0.5	4	0.0170(5)
Sr	0	0	0.35982(7)	0.5	4	0.0170(5)
Mn	0	0	0	0.5	2	0.0200(8)
Zn	0	0	0	0.5	2	0.0200(8)
O1	0.5	0	0	1	4	0.036(2)
O2	0	0	0.1662(5)	1	4	0.034(2)
